# *Arabidopsis* CBF3 and DELLAs positively regulate each other in response to low temperature

**DOI:** 10.1038/srep39819

**Published:** 2017-01-04

**Authors:** Mingqi Zhou, Hu Chen, Donghui Wei, Hong Ma, Juan Lin

**Affiliations:** 1 State Key Laboratory of Genetic Engineering, Institute of Plant Biology, School of Life Sciences, Fudan University, Shanghai 200433, People’s Republic of China

## Abstract

The C-repeat binding factor (CBF) is crucial for regulation of cold response in higher plants. In *Arabidopsis*, the mechanism of CBF3-caused growth retardation is still unclear. Our present work shows that *CBF3* shares the similar repression of bioactive gibberellin (GA) as well as upregulation of DELLA proteins with *CBF1* and *-2*. Genetic analysis reveals that DELLAs play an essential role in growth reduction mediated by *CBF1, -2, -3* genes. The *in vivo* and *in vitro* evidences demonstrate that *GA2-oxidase 7* gene is a novel CBF3 regulon. Meanwhile, DELLAs contribute to cold induction of *CBF1*, -*2, -3* genes through interaction with jasmonate (JA) signaling. We conclude that *CBF3* promotes DELLAs accumulation through repressing GA biosynthesis and DELLAs positively regulate *CBF3* involving JA signaling. CBFs and DELLAs collaborate to retard plant growth in response to low temperature.

Temperature is one of the major environmental factors limiting plant growth. In particular, cold stress is a serious threat to the sustainability of crop yields. While cold extremes during the winter may affect survival, reduced growth at low temperature during the growing season is a key factor limiting plant distribution globally. The changes of ambient temperature affect plant development at multiple points during the lifecycle - from seed germination, plant architecture to flowering and reproductive development. It is crucial that we learn to understand how plants regulate growth in low temperature; this may lead to strategies of manipulating the threshold levels to switch from growth arrest to maintenance of growth. Flowering plants possess a large regulatory network for low temperature responses^1^. In this network, a group of AP2 domain-containing proteins, known as C-repeat (CRT)/Dehydration Responsive Element (DRE) Binding factors (CBF/DREB), plays a crucial role in cold acclimation, an adaptive response that many plant species use to enhance their freezing resistance after an initial exposure to a nonfreezing low temperature^2^.

In *Arabidopsis thaliana*, there are three linearly clustered *CBF1, -2*, -*3* genes^3^,^4^, also known as *DREB1b, DREB1c* and *DREB1a*, respectively, which are identified as key regulators of cold response^5^. Besides, there are three other highly similar genes, *CBF4, DWARF AND DELAYED FLOWERING* (*DDF*)*1* and *DDF2*^6^. *CBF4* is a shared component in both temperature and drought responses^7^ and *DDF1* and *DDF2* are involved in response to high salinity^8^. In addition to freezing response, *CBF1, -2* or *-3* can be rapidly induced by nonfreezing cold stress such as 4 °C or 10 °C and their protein products activate downstream genes known as the CBF regulons, leading to protection of plant cells from low temperature injury^9^. Constitutive expression of *CBF1, -2* or *-3* in *A. thaliana* results in similar effects of increased cold tolerance as well as altered biochemical composition such as proline, glucose, fructose, sucrose and raffinose^10^,^11^,^12^,^13^. At the same time, transgenic plants constitutively overexpressing either *CBF1, -2* or *-3* exhibit similar morphological and developmental phenotypes including stunted growth and delayed flowering, even under non-stressful growth conditions^4^,^14^. The phenomenon of growth retardation caused by the overexpression of *CBF* genes or their homologs has been reported in multiple plant species including those with agricultural importance, such as tomato (*Solanum lycopersicum*)^15^, rice (*Oryza sativa*)^16^, tobacco (*Nicotiana tabacum*)^17^, poplar (*Populus balsamifera*)^18^, potato (*S. tuberosum*)^19^ and peanut (*Arachis hypogaea*n)^20^. Although it is clear that the CBF pathway has a role in affecting plant growth and development, the regulatory mechanism of CBF-caused growth reduction involving downstream genes is uncertain. The fact that both homologous and heterologous expression of *CBF* genes can elicit plant growth repression prevents the effective use of *CBF* genes in molecular breeding. Therefore, the requirement of uncovering how *CBF* genes modulate plant growth under cold stress has been raised.

Previous studies have shown that plant growth regulation during environmental changes is related to phytohormones^14^,^21^. It has been reported that the dwarfism in *CBF1* overexpressing plants including *S. lycopersicum, N. tabacum* and *A. thaliana* can be rescued by exogenous Gibberellin (GA) treatment but not by application of other phytohormones^15^,^17^,^22^. Our previous work also showed that bioactive GA levels were reduced in young leaves of *CbCBF-ox* tobacco and the growth inhibition of *CbCBF-ox* plants was partially due to GA deficiency^23^. These results provide indirect evidence that GA metabolism and signal transduction has a role in CBF-induced plant growth reduction. However, it was also reported that GA treatment could not reverse the growth repression in *CBF3-ox* tobacco (*N. tabacum*)^17^, and effects of GA on *CBF2-ox* plants are still not known. Thus, it is unclear that whether GA has similar interactions with CBF1, -2, -3 transcription factors. In particular, regulatory nodes in this network have yet to be identified, indicating that detailed regulation of GA and *CBF* genes still need to be deeply investigated.

Bioactive GAs promote plant cell elongation^24^,^25^ and are synthesized with the activities of GA20-oxidases^26^ and GA3-oxidases^27^,^28^, but reduced by GA2-oxidases^29^. Bioactive GAs can bind to the Gibberellin Insensitive Dwarf1 (GID1) receptor, and the GA-GID1 complex together with the SCF^SLY1^ E3 ligase facilitate ubiquitination of DELLA proteins and their subsequent degradation by the 26S proteasome^30^,^31^. DELLAs are the master negative regulators of the GA signaling and their abundance will lead to severe growth restriction^32^,^33^,^34^,^35^,^36^. There are five DELLAs [REPRESSOR of gal-3 (RGA), GA INSENSITIVE (GAI), RGA-LIKE1 (RGL1), RGL2, and RGL3] in *A. thaliana*, which display overlapping but non-identical functions in repressing GA responses^37^. Although it is known that both the cold-induced CBFs and GA signaling pathways regulate plant growth and stress tolerance, it is unclear whether and how these pathways directly interact with each other.

The goals of this study were to better understand the crosstalk between GA signaling and CBF3. We have tested the bioactive GA levels and DELLA accumulation in *cbf3* knock-out mutant and *CBF3-ox* plants, uncovering the positive role of *CBF3* in DELLA modulation. Meanwhile, we have also shown the contribution of DELLAs in cold induction of *CBF3* through interaction with jasmonate (JA) signaling. Our results clarify the role of CBF3 in the interplay with GA signaling and identify *GA2ox7* as a novel CBF3 regulon.

## Results

### CBF3 mediates cold induced reduction of gibberellin level and plant growth retardation

**Figure 1:**
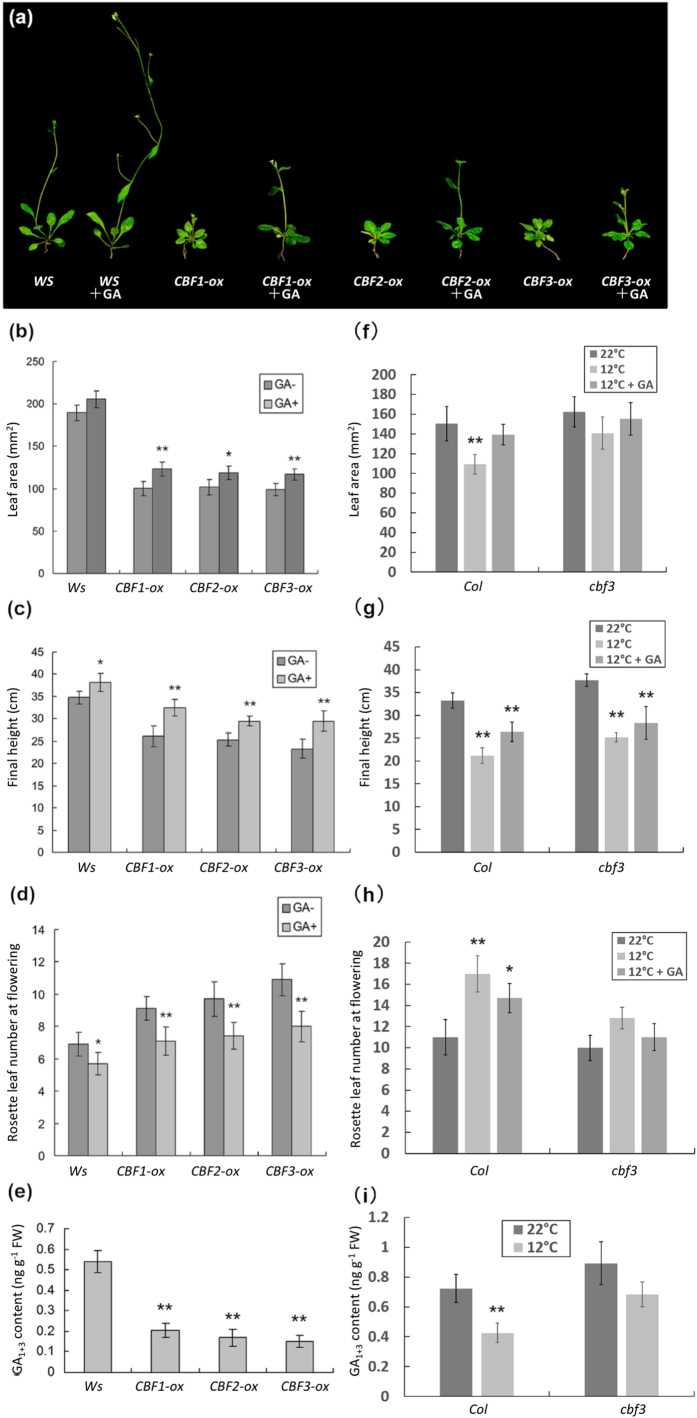
CBF3 suppresses plant growth through negative regulation of bioactive GA level and GA reduction in low temperature is mediated by CBF3. (**a**) Representative phenotypes of 4-week-old *CBF1-ox, CBF2-ox* and *CBF3-ox* plants with or without GA_3_ application. Dwarfism caused by *CBF1, -2, -3* overexpression can be partially rescued by 10^−5^ M GA_3_ application. Phenotypes including (**b**) the areas of fifth rosette leaves, (**c**) the final heights, (**d**) the rosette leaf numbers and (**e**) GA_1+3_ contents are shown. In *cbf3* mutant cold induced growth repression and GA reduction are weakened according to (**f–h**) growth phenotypes and (**i**) GA_1+3_ contents. (SE, n = 20, *P < 0.05, **P < 0.01).

In *A. thaliana*, dwarfism of GA-deficient mutant *ga1-3* can be reversed by the treatment of GA_3_^38^, while GA-insensitive mutant *gai* cannot^39^. To determine whether the growth retardation phenotypes caused by increased *CBF3* resemble GA-deficient or GA-insensitive mutants, we investigated the GA_3_ response of *CBF1*-*ox, CBF2*-*ox* and *CBF3*-*ox* plants. We treated *CBF1*-*ox, CBF2*-*ox* and *CBF3*-*ox* seedlings with GA_3_ both in MS plates and in soil. Interestingly, *CBF1*-*ox, CBF2*-*ox* and *CBF3*-*ox* plants exhibited similar phenotypes under low concentration of GA_3_ treatments (Fig. 1a; Fig. S1). Growth retardation caused by *CBF3* in plant height and flowering time were restored to WT (wild type control) level and leaf area was also partially restored (Fig. 1b–d), which was similar to the effects of *CBF1, -2* here as well as previously reported instances of *CBF1* and *DDF1*^22^,^40^. Next, we tested the endogenous bioactive gibberellin level in 4-week-old *CBF1*-ox, *CBF2*-ox and *CBF3*-ox plants. Consistently, GA_1+3_ levels of these plants were all significantly decreased (Fig. 1e). These suggested that *CBF1, -2* or *-3* genes similarly downregulate GA level in cold response. In particular, *cbf3* mutant showed weaker growth reduction in leaf size and flowering time under low temperature, and these two indices of *cbf3* were close to that of GA_3_ rescued *Col* plants at 12 °C (Fig. 1f,h). For plant height, no obvious difference between *Col* and *cbf3* was observed, indicating that height can be affected by CBF3-independent pathways (Fig. 1g). Further, *cbf3* showed less reduction of GA_1+3_ levels compared with *Col* in response to chilling temperature (Fig. 1i). Together, CBF3 participates in the control of GA repression and restrained growth in the face of cold stress.

### CBF3 represses plant growth through DELLA accumulation under low temperature

**Figure 2:**
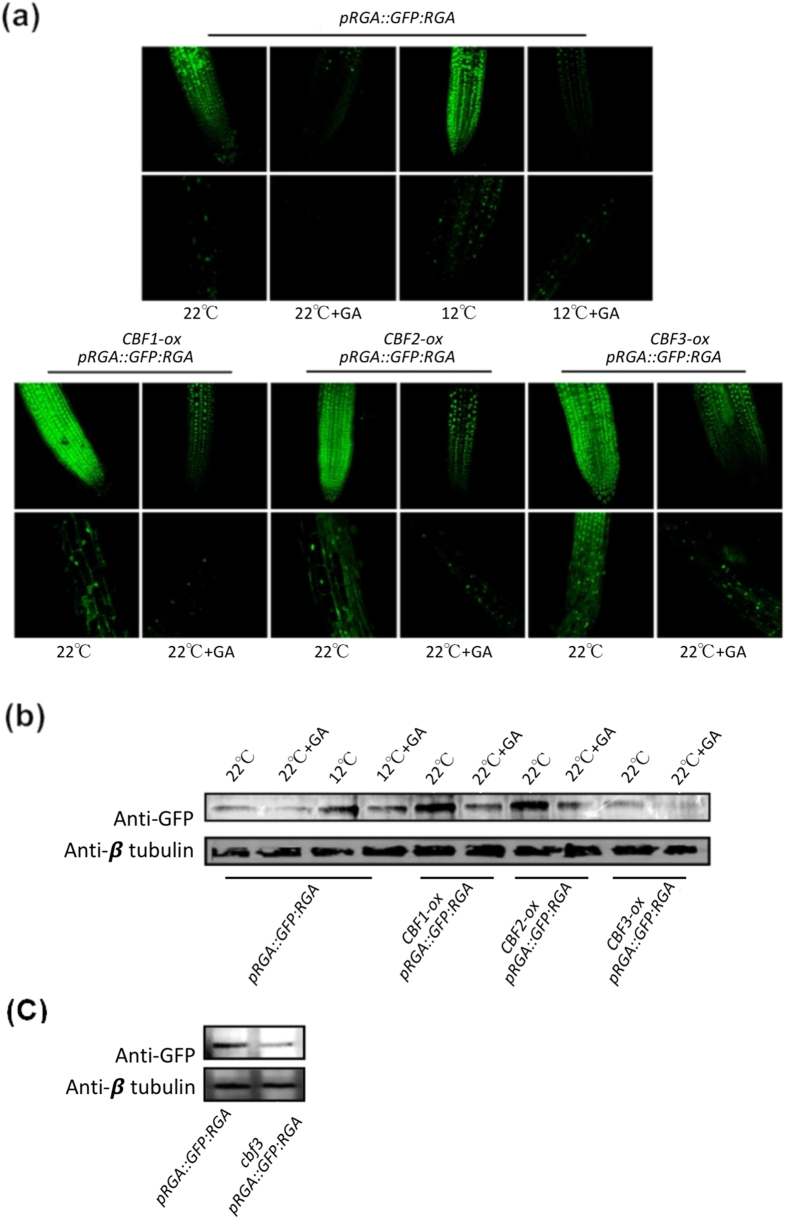
*CBF3* enhances DELLA accumulation in low temperature and do not affect GA-mediated DELLA degradation. (**a**) GFP fluorescence in the root tip (first row) and elongating zone (second row) of 8-day-old of *pRGA::GFP:RGA, CBF1-ox pRGA::GFP:RGA, CBF2-ox pRGA::GFP:RGA* and *CBF3-ox pRGA::GFP:RGA* seedlings. Images are taken with identical parameters for comparison of fluorescence levels. (**b**) Immunoblot analysis of GFP:RGA levels in leaves from 4-week-old plants indicated. (**c**) GFP:RGA levels in 8-d-old seedlings treated at 12 °C for 4 h. The β-tubulin is used as loading control.

**Figure 3:**
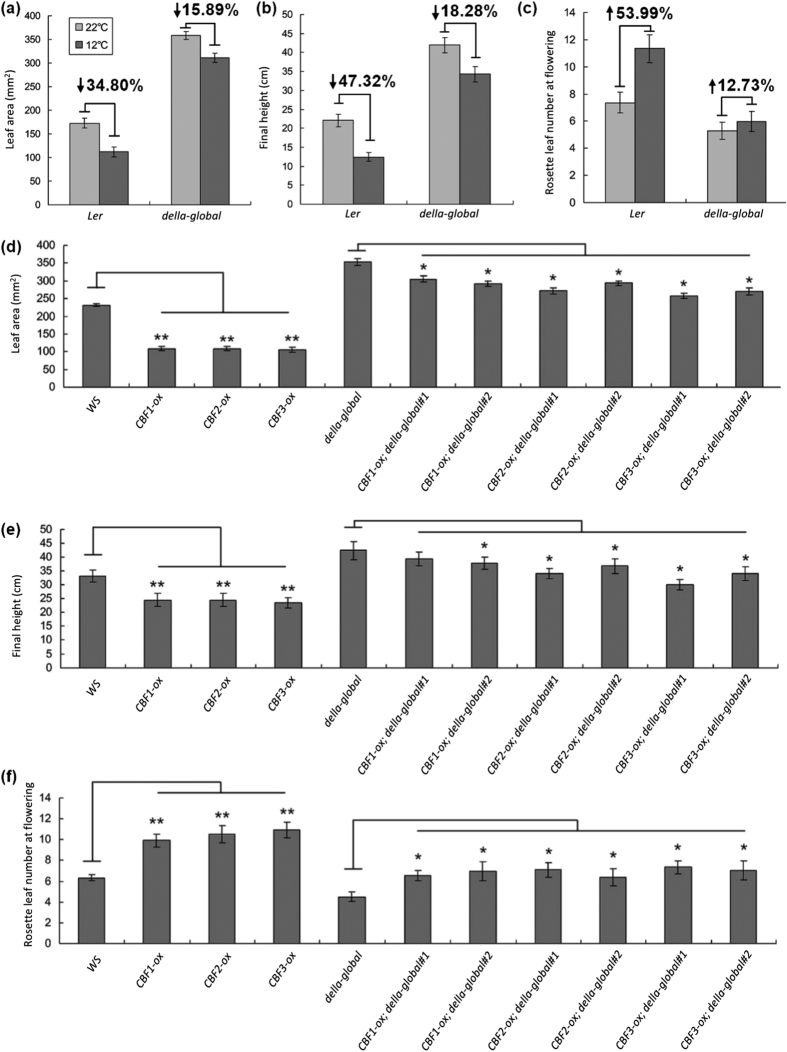
The *della-global* mutation weakens growth retardation caused by low temperature and *CBF1, -2, -3* overexpression. (**a–c**) Comparison of *Ler* and *della-global* plants. Up or down arrows represent increase or decrease relative to wild type, respectively. (**d–f**) Comparison between *Ws* and *CBF1-ox, CBF2-ox* and *CBF3-ox* plants as well as comparison between *della-global* and *CBF1-ox della-global* plants, *CBF2-ox della-global* plants, *CBF3-ox della-global* plants. (SE, n = 20, *P < 0.05, **P < 0.01).

DELLA proteins are key growth inhibitors that can be accumulated in GA-deficient plants^36^. Since CBF3 reduced gibberellin levels, we assumed that it was also involved in DELLA regulation. According to the report that late flowering of *A. thaliana* at a low temperature of 12 °C could be obviously restored in *della-global* mutants^41^, we tested GFP:RGA fusion protein levels in plants with or without GA_3_ application at 22 °C or 12 °C. The GFP:RGA level was obviously enhanced at 12 °C as well as *CBF1-ox, CBF2-ox* and *CBF3-ox* background in 8-day-old roots (Fig. 2a). In 4-week-old leaves, similar elevation of GFP:RGA level was observed (Fig. 2b). The *CBF3-ox* plants showed a lower level of GFP:RGA compared with *CBF1-ox* and *CBF2-ox* in normal temperature, suggesting that in late growth stage CBF1 and CBF2 may have stronger effects in RGA level than CBF3. Moreover, GFP:RGA level was lower in *cbf3* mutant than *Col* under low temperature, indicating the positive role in modulating RGA level of CBF3 (Fig. 2c). On the other hand, GA_3_ leaded to degradation of GFP:RGA both under cold condition and in *CBF1-ox, CBF2-ox, CBF3-ox* plants, suggesting that *CBF1, -2* and *-3* may not affect GID1 and SLY1 function. Next, to confirm the contribution of DELLAs to growth repression caused by *CBF1, -2* or *-3*, we created transgenic plants that constitutively express *CBF1, -2* or *-3* in *della-global* (*gai-t6*; *rga-t2*; *rgl1-1*; *rgl2-1*; *rgl3-1*) background. Two lines with high transgenic expression level for each were used for further analysis (Fig. S2). Consistent with the study of Kumar *et al*.^41^, *della-global* mutation significantly weakened the growth retardation at 12 °C (Fig. 3a–c). Similar restoration was observed in *CBF1-ox della-global, CBF2-ox della-global* or *CBF3-ox della-global* plants (Fig. 3d–f and Fig. S3). The differences in leaf area, plant height and leaf number at flowering were strongly reduced by *della-global* mutation. These demonstrated that *CBF3* inhibits plant growth through accumulating DELLAs under low temperature.

### CBF3 upregulates the DELLA and *GA2ox* genes expression

**Figure 4:**
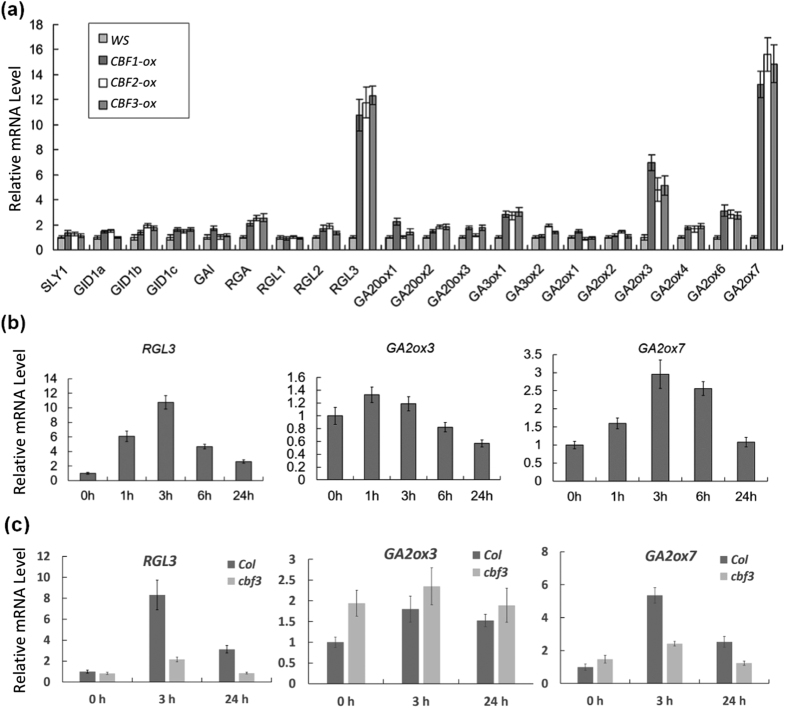
CBF3 increases transcript levels of *RGL3, GA2ox3* and *GA2ox7*. (**a**) Relative expression levels of GA metabolism and signaling genes in *Ws* and *CBF1-ox, CBF2-ox* and *CBF3-ox* plants. (**b**) Relative expression levels of *RGL3, GA2ox3* and *GA2ox7* in *Ws* plants under 12 °C treatment. (**c**) Relative expression levels of *RGL3, GA2ox3* and *GA2ox7* in *Col* and *cbf3* plants under 12 °C treatment. Data are means ± SE.

The GAs level in plants is homeostatically modulated through GA biosynthesis and deactivation pathways, two processes catalyzed by three categories of dioxygenases, which are respectively encoded by a small gene family^42^. GA 20-oxidases (GA20ox) and GA 3-oxidases (GA3ox) catalyze successive steps in the synthesis of bioactive GAs^27^, while GA 2-oxidases (GA2ox) deactivate bioactive GAs^29^. To further figure out the mechanism involved in GA decrease and DELLA increase, we tested the expression pattern of GA signaling and metabolic genes in *CBF1-ox, CBF2-ox* and *CBF3-ox* plants. Similar to *CBF1-ox* and *CBF2-ox* plants, *CBF3-ox* lines showed significantly higher transcript levels of *RGL3, GA2ox3* and *GA2ox7* than *Ws* plants (Fig. 4a). Meanwhile, *RGL3* and *GA2ox7* could also be induced by cold treatment, while *GA2ox3* was slightly affected in *Ws* plants (Fig. 4b), suggesting that *GA2ox3* might be affected by other regulators under low temperature. In addition, no *GA20ox* or *GA3ox* genes were repressed and *RGA, GA20ox1, GA3ox1* and *GA2ox6* expression were slightly enhanced between two and four folds in *CBF1-ox, CBF2-ox, CBF3-ox* lines (Fig. 4a). Previous work reported that *GA20ox* and *GA3ox* transcripts could be increased by DELLA accumulation due to a feedback mechanism^26^,^29^. In *Col* background, cold induction pattern of *RGL3, GA2ox3* and *GA2ox7* were similar to *Ws* and *cbf3* mutation blocked elevation of *RGL3* and *GA2ox7* transcript levels in cold treatment (Fig. 4c). Interestingly, *GA2ox3* had even a higher transcript level at 22 °C in *cbf3* plants and showed a similar expression level in cold condition compared with WT, implying that *GA2ox3* may be downregulated by CBF3 in the normal condition and CBF3 is not required for expression of *GA2ox3* at low temperature. The enhancement of *GA2ox3* expression in *CBF3-ox* plants can be due to indirect feedback mechanisms. In a word, CBF3 confers a transcriptional increase of *RGL3* and *GA2ox7* gene, which is consistent with the altered GA and DELLA levels.

### *GA2ox7* is a CBF3 regulon

**Figure 5:**
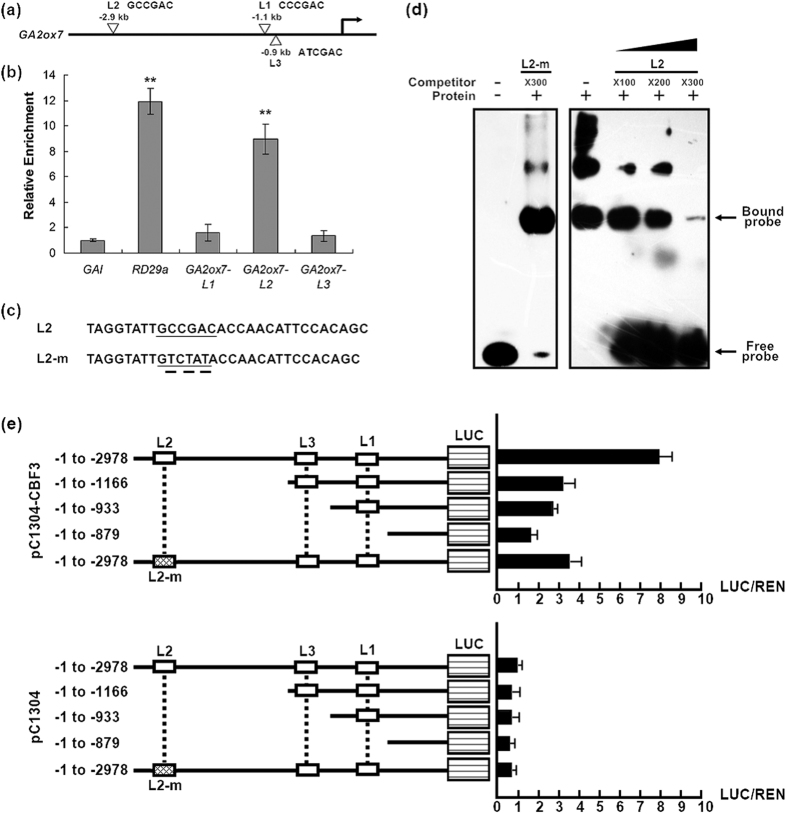
*GA2ox7* is a CBF3 regulon. (**a**) Schematic diagram of three CRT/DRE-like elements in the promoter region of *GA2ox7*. Core sequences of L1, L2 and L3 are shown. (**b**) ChIP qRT-PCR analysis of CBF3 binding to the three CRT/DRE-like elements of *GA2ox7*. The −0.2 kb to −0.35 kb promoter region of *RD29a* containing three CRT/DRE-like elements serves as positive control and one area without CRT/DRE-like elements in the CBF-noninduced gene *GAI* is used as negative control. Data are means ± SE. (**c**) Oligonucleotides of L2 and L2-m (mutated version) elements within the *GA2ox7* promoter used in the EMSA. Underlined letters are core sequences of CRT/DRE. Three nucleotides are substituted in L2-m. (**d**) CBF3 binds to L2 element of *GA2ox7* promoter *in vitro*. Unlabeled L2 and L2-m elements fragment are used as competitors. (**e**) Dual-LUC Assays using transient expression system in tobacco leaves. CBF3 driven by 35S promoter was served as the effector and LUC under control of *GA2ox7* promoter truncations as indicated were reporters. The relative activity (LUC/REN) were shown. Reporters co-transformed with the blank pC1304 vector were used as controls. Data are means ± SE.

Since *GA2ox7* and *RGL3* were significantly induced by *CBF3* overexpression, we decided to test whether they were the targets of CBF3. The binding sequence of CBF transcription factors in promoter regions is defined as CRT/DRE element with a core sequence of A/GCCGAC^43^,^44^,^45^. In the presumed promoter region of −0.2 kb to −0.35 kb from the initiation codon of *RD29a*, a well-known CBF regulated gene, there are three ACCGAC and one GCCGAC motifs. Thus this area can be a good positive control in ChIP-qPCR for detection of *in vivo* binding of transcription factor to the promoter. Meanwhile, one region without CCGAC in the *GAI* gene not induced by CBF was used as a negative control. There are three putative CRT-like elements (designated L1, L2 and L3) in −0.9 kb to −2.9 kb regions of *GA2ox7* and among them only L2 has the exact CRT/DRE core sequence (GCCGAC) (Fig. 5a). Consistently, we observed the enrichment of CBF3 near L2 but not L1 or L3 according to ChIP-qPCR (Fig. 5b). Besides, there is no exact CRT/DRE core sequence in *RGL3* promoter regions. We identified two similar elements with sequence of GTCGAC in −0.74 kb to −0.76 kb region of *RGL3* instead. However, no recruitment of CBF3 was detected in these areas (data not shown).

Subsequently we also used EMSA to confirm the binding of CBF3 to L2. The DNA fragments containing L2 or mutated version L2-m were used as probes (Fig. 5c). The 5′ biotin-labeled L2 was incubated with CBF3-His protein and several complexes were observed (Fig. 5d). Without competitors, all probes were bound to CBF3-His protein. Addition of increasing amounts of cold competitors with the same sequence weakened the complexes and released free probes, while competitor with mutated sequence did not abolish probe-bound complex bands, suggesting the specificity of the binding in the CBF3-L2 complex (Fig. 5d). However, the concentration of cold competitors needed for eliminating probe-bound complex bands was high (x300) and the binding affinity of CBF3 to L2 appeared to be somewhat low, which could be due to the *in vitro* reaction condition. For further validating the activation of *GA2ox7* regulated by CBF3, we performed the *in vivo* dual-LUC assay using transient expression of CBF3 driven by 35S promoter (used as the effector) and LUC driven by truncated *GA2ox7* promoter fragments (used as reporters) (Fig. 5e). The *GA2ox7* promoter reporter containing L2 + L3 + L1 that was co-transformed with CBF3 showed highest relative LUC/REN activity. The fragments of L3 + L1 or L1 lacking L2 moderately upregulated LUC, which was nearly in a half level of L2 + L3 + L1 induction, and the truncation excluding all three elements showed lowest LUC intensity. Interestingly, the promoter harboring mutated L2 (L2m + L3 + L1) exhibited the LUC/REN ratio that was similar to L3 + L1 or L1, demonstrating the contribution of L2 in the activation of *GA2ox7* by CBF3. The control groups without CBF3 all showed extremely low activity of LUC, indicating the weak basal transcription of *GA2ox7*. These verified the *in vivo* activity of CBF3 in the induction of *GA2ox7,* which is consistent with the qPCR results. Together, ChIP and EMSA analyses suggested the interaction of CBF3 and *GA2ox7* promoter, and LUC assay indicated the function of CBF3 in transcriptional regulation of *GA2ox7*. We propose that *GA2ox7* is a newly identified CBF3 regulon that can be upregulated by CBF3 through the CRT/DRE element in plants.

### DELLAs contribute to cold induction of *CBF3*

**Figure 6:**
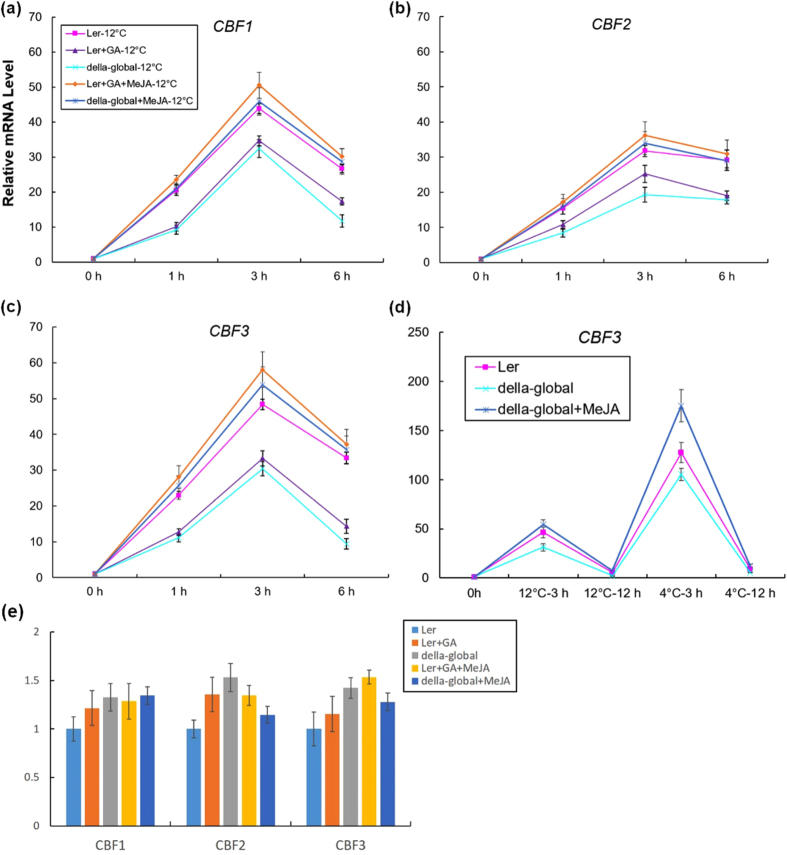
DELLAs contribute to cold induction of *CBF1, -2, -3* through interaction with JA signaling. (**a–c**) Altered cold induction levels of *CBF1, -2, -3* in *Ler* and *della-global* plants under GA_3_, MeJA, GA_3_ together with MeJA or 0.1% ethanol treatments. (**d**) Expression level of *CBF1, -2, -3* in *Ler* and *della-global* plants under GA_3_ or MeJA treatments at 22 °C. (**e***) CBF3* expression level under 12 °C and 4 °C in seedlings indicated. Data are means ± SE.

Interplay between GA and cold responsive signaling raises the question of how DELLAs regulate CBFs. The fact that *CBF* genes are transiently induced to a peak after around 3 h of cold application and DELLAs accumulation stays in a high level decreases the possibility that DELLAs directly target *CBF* genes. Indeed, no DELLA binding activity was detected in *CBF* gene regions. It has been reported that MeJA modulates CBF signaling through degradation of JASMONATE ZIM-domain (JAZ)s, a repressor of INDUCER OF CBF EXPRESSION 1 (ICE1)^46^. ICE1 plays a central role in *CBF3* cold induction. At the same time, DELLAs can regulate JA signaling via interaction with JAZs to release MYC2, a key transcription activator in JA signaling^47^,^48^. Since the direct bindings between DELLAs and JAZs as well as JAZs and ICE1 have been revealed, ICE1 can also be released from JAZs binding by DELLAs and strongly induce CBF3 expression when cold temperature comes down. As the next step, to investigate the potential regulation of CBFs by DELLAs we measured cold induction of *CBF1, -2, -3* genes when GA_3_ and MeJA were applied. Compared with WT, the cold induction of three *CBF* genes were all significantly weaker in *della-global* mutants. Same changes happened when MS medium contained 10^−5^ M GA_3_ (Fig. 6a–c). Nevertheless, when MeJA was present, influence by GA_3_ treatment or DELLA mutation were eliminated and the cold induced transcript levels of *CBF1, -2, -3* were even higher than control. Likewise, after a transient induction at 12 °C *CBF3* showed a higher peak under 4 °C treatment and MeJA application enhanced this induction while *della-global* mutation partially blocked it (Fig. 6d). Coordinately, JA positively regulates DELLAs accumulation according to increased GFP:RGA (Fig. S4) and RGL3-GFP levels after MeJA treatment^48^. Interestingly, without cold treatment at 22 °C, *CBF1, -2, -3* did not have obvious difference of expression level in comparison between *Ler* and *della-global* seedlings with these kinds of GA_3_ or MeJA application (Fig. 6e), suggesting that GA_3_ or MeJA might not affect *CBF1, -2, -3* in normal temperature. These demonstrated that DELLAs played a positive role in cold induction of *CBF1, -2, -3* involving JA signaling, which was consistent with our hypothesis.

## Discussion

**Figure 7:**
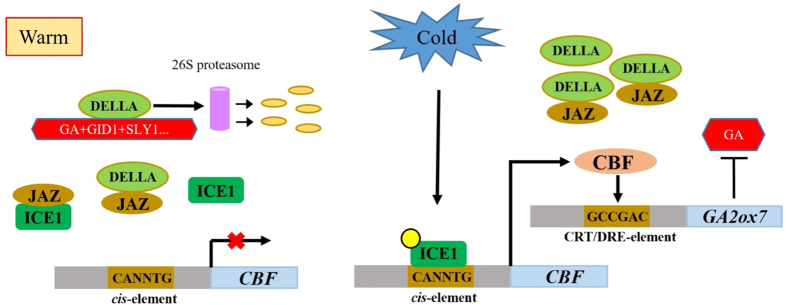
Model for positive regulation between CBF3 and DELLAs in response to low temperature. In warm temperature, DELLAs interact with JAZs to prevent JAZs binding to ICE1. Meanwhile, DELLAs are degraded through GA mediated signaling. In cold temperature, ICE1 is modified to gain the function for activation of *CBF3* transcription. CBF3 activates *GA2ox7* to decrease the bioactive GA level and subsequently promotes the accumulation of DELLAs. Increased DELLAs release more ICE1 to enhance next round of *CBF3* cold induction.

The CBF signaling pathway is conserved in higher plant species^14^. For modulation of freezing tolerance and cold acclimation, overexpression of three *CBF* genes in *A. thaliana* results in enhanced freezing tolerance^49^, whereas *cbf1* or *cbf3* loss-of-function single mutant increases plant sensitivity to freezing stress after cold acclimation^50^, the *cbf2* mutant shows a freezing tolerance phenotype with or without cold acclimation^51^. These results indicate CBF1 and CBF3 play a different role than CBF2^50^,^51^. For growth restriction and late flowering, phenotype caused by *CBF1* overexpression is mainly mediated by GA/DELLA signaling^22^. The dwarfism conferred by *CBF3* and *CBF2* would appear to involve either different mechanism(s) or same from that reported for *CBF1*. Here we determine the matching function of *CBF1, -2*, -*3* involved in the crosstalk with GA/DELLA signaling and cause of growth reduction, in agreement with the analysis in *CBF1-ox, CBF2-ox, CBF3-ox* lines^4^,^13^. Increased *CBF3* expression level has the same effects compared with *CBF1* and *CBF2* according to decreased GA levels and abundant DELLA proteins. Meanwhile, DELLAs play a positive role in cold induced expression of *CBF1, CBF2* and *CBF3* through interacting with JA signaling (Fig. 7). Recent reports also confirmed that *CBF1, CBF2* and *CBF3* transcription factors regulate very similar gene sets^52^. Contrary to our results, Kasuga *et al*.^17^ showed that 10^4^ M GA_3_ caused no reversal of growth reduction in *CBF3-ox* tobacco plants and only enlarged leaf area under GA_3_ application was observed in *CBF3-ox Arabidopsis*. Moreover, Cong *et al*.^53^ also reported that *CBF3-ox* tobacco leaves were enlarged and petioles were lengthened by 10^−4^ M GA_3_^53^. In our case, lower concentration of GA_3_ (10^−5^ M and 10^−6^ M) promotes growth in both WT and transgenic plants; nevertheless, the percentages of changes in transgenic plants are significantly higher (Fig. 1b–d). We also show that three kinds of overexpression Arabidopsis plants have a similar response to continuous application of GA_3_ on plates. These indicate that different treatment conditions including concentration of GA_3_, treatment timing or time duration can lead to diverse phenotypes in different plant materials.

Consistent with some previous reports, we also show that *RGL3* can be significantly induced by *CBF1, -2* and *-3* overexpression^22^,^54^. Surprisingly, no binding activity of CBF3 protein is detected in the putative CRT/DRE-like elements of *RGL3* promoter in our assay. It has been acknowledged that CBF proteins do not bind equally to all CRT/DRE-like elements^51^,^52^. In any case, the induction of *RGL3* by CBFs could be indirect. In other DELLA genes or GA metabolic genes, no CRT/DRE-like elements are observed^8^ and the only identified regulatory node in the crosstalk between CBFs and DELLAs is *GA2ox7*. It has been reported that DDF1, a homolog of CBFs in *A. thaliana* that regulates high-salinity response, also binds to promoter region of *GA2ox7* and therefore reduces GA level^45^. Thus *GA2ox7* can be a key component of CBF/DREB1 signaling pathway modulating GA and DELLAs in response to multiple abiotic stresses, which is a good candidate of modification target in the genetic engineering and molecular breeding of stress tolerant crops without yield penalty.

There is an evidence supporting that the activation of CBF regulons by CBF1 is in a DELLA-independent fashion - when CBF1 was overexpressed in normal temperature, transcript levels of CBF regulons are similar in WT and DELLA mutants^22^. The present work reveals that although DELLAs do not affect CBFs regulation in CBF regulons transcription, they contribute to the cold induced expression of *CBF1, -2*, -*3* instead. The *CBF* genes expression in warm temperature and cold induction are in different regulatory routes. ICE1, the key activator of *CBF* genes in cold induction, is constitutively expressed and can only be modified to gain function for activation of *CBF* genes under low temperature^2^,^46^. Previous analysis of DELLA direct targets did not detect binding of DELLAs to *CBF1, -2*, -*3* promoter regions^55^, thus DELLAs can regulate cold induction of *CBF1, -2*, -*3* through ICE1. Recent studies on JA signaling provide a clue connecting ICE1, JAZs and DELLAs. JAZs, a major repressor in JA signaling, directly targets ICE1 to inhibit the activation of CBFs, while DELLAs competitively bind to JAZs to release MYC2 to activate JA response^46^,^47^,^48^. Meanwhile, MYC2 also interacts with ICE1 to enhance *CBF* genes transcription in cold condition^56^. Indeed, our work shows that MeJA treatment, which degrades JAZs and activates MYC2, eliminates the effects from GA and *della-global* mutation and increases *CBF1, -2*, -*3* cold induced expression levels (Fig. 6a–d). Notably, due to the transient induction of *CBF1, -2*, -*3* under low temperature, the abundance of DELLAs caused by CBFs unlikely have a direct feedback to induce CBFs. Consistently, neither GA nor JA change *CBF1, -2*, -*3* expression in warm temperature, suggesting that DELLAs strengthen “priming” of induction of *CBF* genes before cold application through JA-dependent pathway. When DELLAs are abundant, the subsequent induction of *CBF* genes can be enhanced (Fig. 7).

In addition, the present work shows that GA application or *della-global* mutation does not completely recover the growth repression of *CBF1-ox, CBF2-ox* or *CBF3-ox* plants, especially for *CBF3-ox* seedlings. Hence there are still some DELLA-independent pathways involved in CBF-caused growth retardation. Analysis in a *CBF* gene from *Capsella bursa-pastoris* revealed that *CbCBF* also affected cell cycle signaling besides antagonizing with GA^23^. In *A. thaliana*, although microarray has been used in some work about CBF signaling, more powerful tools such as deep RNA-seq will be needed to uncover more details. In summary, the present knowledge of positive regulation between DELLAs and CBF transcription factors as well as the investigation in additional unknown mechanism of how CBFs restrain growth can contribute to the accurate genetic control in molecular breeding of tolerant crops.

## Methods and Materials

### Plant materials and treatments for phenotyping

The *A. thaliana* seeds were grown in pots at 22 °C under 16-h-light/8-h-dark cycle. The *cbf3* knock-out line (SAIL_244_D02)^57^, *della-global* mutant (*gai-t6*; *rga-t2*; *rgl1-1*; *rgl2-1*; *rgl3-1*)^58^ and *pRGA::GFP:RGA* line (*Col* and *Ler*)^59^ were obtained from Arabidopsis Biological Resource Center. To generate *CBF1*-*ox della-global, CBF2*-*ox della-global, CBF3*-*ox della-global, CBF1*-*ox pRGA::GFP:RGA, CBF2*-*ox pRGA::GFP:RGA* and *CBF3*-*ox pRGA::GFP:RGA* plants, the full length of the *CBF1, CBF2* or *CBF3* coding sequence was cloned into pCAMBIA1304 vector using primers listed in Table S1 and transformed into *della-global* and *pRGA::GFP:RGA* plants. The *cbf3* knock-out line (SAIL_244_D02) was crossed with *pRGA::GFP:RGA* line (*Col*) and *cbf3 pRGA::GFP:RGA* line was isolated from F3 progeny. *CBF1-ox, CBF2-ox* and *CBF3-ox* plants in *Ws* background were previously described^4^. For phenotyping in low temperature, 14-d-old plants growing at 22 °C were transferred to 12 °C and applied to the measurements when they were 4-week-old. Gibberellin spray treatments were performed as previously described^23^. For phenotyping on plates, GA_3_ with concentration of 10^−5^ and 10^−6^ M was added to Murashige and Skoog (MS) agar medium and seedlings were grown at 22 °C for 3 weeks. For leaf area analysis, the fifth rosette leaves of 4-week-old plants were collected and determined for size with IMAGEJ (http://rsbweb.nih.gov/).

### Measurements of endogenous gibberellin contents in *Arabidopsis*

The endogenous gibberellin level in *A. thaliana* was measured using enzyme linked immunosorbent assay (ELISA) as described elsewhere^23^. Briefly, samples were extracted in cold 80% (v/v) methanol with 1 mM butylated hydroxytoluene overnight at 4 °C. After centrifugation at 10, 000 g for 20 min, the extracts were passed through a C_18_Sep-Pak cartridge (Waters, Milford, MA, USA) and residues were dissolved in 10 mM PBS buffer (pH 7.4). Meanwhile, the 96-well microtitration plates (Nunc, Denmark) was coated with synthetic GA_1_-ovalbumin conjugates in 50 mM NaHCO_3_ buffer (pH 9.6) overnight at 37 °C. Samples were incubated with HRP-labeled goat anti-rabbit immunoglobulins for 1 h at 37 °C and ovalbumin solution (10 mg/mL) was used to block nonspecific binding. The enzyme-substrate reaction was carried out in the dark and data were calculated according to absorbance of 490 nm. The cross-reactivity of antibodies raised against GA_1_-ovalbumin to GA_3_-ovalbumin was 32% based on previous report^60^.

### Cold and phytohormone treatments for transcript and protein level tests

For gene expression tests in *Ws* and *Col* plants, 14-d-old seedlings growing in soil at 22 °C were transferred to 12 °C and rosette leaves were collected. For *Ler* and *della-global* plants, 4-d-old seedlings grown in MS medium containing 10 μM GA_3_, 5 μM MeJA^46^, GA_3_ together with MeJA or 0.1% ethanol at 22 °C were transferred to 12 °C. For *pRGA::GFP:RGA* lines, 8-d-old seedlings were incubated in 50 mM MeJA^48^ for time designed. The plant materials were collected immediately in liquid nitrogen at each time point of treatments as indicated and stored at −80 °C until use.

### Quantitative real-time PCR

Total RNA was extracted using Plant RNA Mini Kit (Watson Biotechnologies, Inc, China). RNA concentration was estimated by spectrophotometer (WFZUV-2100, Unico^TM^ Instruments Inc.) and genomic DNA was removed using DNAase I (Promega, Madison, WI, USA). Approximately 1 μg RNA was reverse transcribed using PrimeScript^®^ RT Master Mix (Takara, China) at 37 °C for 20 min. The PCR amplification reactions were carried out using SYBR^®^ Premix Ex Taq™ II (Perfect Real Time) (Takara, China) with three replicates for each sample and the *Actin2* gene (AY096381) was used as internal control. The 2^−△△Ct^ method was used to determine the relative mRNA abundance and primers used in this work are all listed in Table S1.

### Confocal Microscopy analysis

The 8-d-old seedlings of *pRGA::GFP:RGA, CBF1-ox pRGA::GFP:RGA, CBF2-ox pRGA::GFP:RGA* and *CBF3-ox pRGA::GFP:RGA* grown on MS plates were sprayed with 10^−5^ M GA_3_ or 0.1% ethanol at 22 °C or 12 °C, respectively. After 4 h the root tip and elongating zone were excised with razor blade. GFP:RGA fusion protein level was detected by confocal laser scanning microscopy (Leica TCS NT, Wetzlar, Germany) as mentioned previously^61^.

### Protein extraction and western blot

Proteins were extracted using Plant Protein Extraction Reagent kit (CWBIO, China). Approximate 1 g plant tissues were used for each sample. Protein samples were separated by SDS-PAGE on 10% polyacrylamide gels and transferred onto PVDF membranes according to standard protocols. Membranes were probed with anti-GFP antibody (Beyotime Biotechnology, China) at a 1:2000 dilution and signals were visualized on a Typhoon system (GE Healthcare, http://www.gelifesciences.com/).

### Electrophoretic mobility shift assay

*CBF3* cDNA was cloned into pET-28a vector using primers CBF3-HisF and CBF3-HisR (Table S1). Soluble CBF3-His protein was expressed in *Escherich coli* strain BL21 and purified using HisPur™ Cobalt Resin (Thermo, USA). The 5′ biotin-labeled double-stranded oligonucleotides GA2ox7-L2 was used as a probe while non-labeled GA2ox7-L2 and GA2ox7-L2-m (mutated) were used as competitors, respectively. The labeled probes (3 pM) with or without unlabeled competitors were incubated for 20 min at room temperature with 4 μg of purified CBF3-His fusion protein in binding buffer (25 mM HEPES-KOH buffer at pH 7.9, containing 50 mM KCl, 0.5 mM EDTA, 0.5 mM DTT and 10% glycerol) supplemented with 20 pM poly (dI-dC). The resulting DNA-protein complexes were resolved by electrophoresis on a 6% non-denaturing polyacrylamide gel in 0.5 × TBE buffer and transferred by electroblotting to PVDF membranes. After crosslinked under UV (120 mJ/cm^2^, 254 nm), the signal was visualized according to manufacturer instruction of LightShift Chemiluminescent EMSA kit (Thermo, USA).

### Chromatin Immunoprecipitation

Chromatin immunoprecipitation was performed as previously described^55^. Briefly, 30 μL protein A-agarose beads (Epigentek) were incubated with 5 μg anti-CBF3 antibody at 4 °C for overnight. Soluble CBF3-His protein described above was used to immunize two rabbits to obtain antiserum. The immunoglobulin G (polyclonal antibodies) fraction was purified by affinity chromatography using protein G-agarose (Sigma-Aldrich, USA). Preserum was used as a background control. Around 3 g of washed 4-week-old plants were submerged in 50 mL of crosslinking buffer (10 mM Tris-HCl, pH8, 0.4M Suc, 1 mM PMSF, 1 mM EDTA and 1% formaldehyde) and vacuum infiltrated for 5 min at room temperature. The cross-linking was stopped by transferring plant materials to 250 mM Gly and vacuum infiltration for 5 min. Plant tissues were ground in liquid nitrogen and resuspended in 25 mL cold nuclei isolation buffer (15 mM PIPES, pH 6.8, 0.25 M Suc, 5 mM MgCl_2_, 60 mM KCl, 15 mM NaCl, 1 mM CaCl_2_, 1% Triton X-100, 20 mM sodium butyrate, 1 mM PMSF, 2 μg/mL pepstatin A and 2 μg/mL aprotinin). The homogenized slurry was filtered and centrifuged at 3800 g for 20 min to get the pellet (nuclei). The nuclei were resuspended in 1.5 mL of lysis buffer (50 mM HEPES, pH 7.5, 150 mM NaCl, 1 mM EDTA, 0.1% SDS, 0.1% sodium deoxycholate, 1% Triton X-100, 20 mM sodium butyrate, 1 μg/mL pepstatin A and 1 μg/mL aprotinin). DNA was sheared into 200 bp to 1000 bp fragments by 6–10 min of 5 sec pause sonication at 40% amplitude using a TekMar TM-100 sonic disruptor (TekMar). After centrifugation at 13,800 g for 10 min, the supernatant was diluted for five folds with nuclei lysis buffer. The prepared mixture of protein A-agarose beads and antibody was added and the sample was incubated at 4 °C for overnight with gentle rotation. After 3800 g centrifugation for 2 min, the agarose beads were sequentially washed with low salt wash buffer (20 mM Tris-HCl, pH 8, 150 mM NaCl, 0.2% SDS, 0.5% Triton X-100, 2 mM EDTA), high salt wash buffer (20 mM Tris-HCl, pH 8, 500 mM NaCl, 0.2% SDS, 0.5% Triton X-100, and 2 mM EDTA), LiCl wash buffer (10 mM Tris-HCl, pH 8, 0.25 M LiCl, 1% sodium deoxy-cholate, 1% Nonidet P-40, and 1 mM EDTA), and TE buffer (twice; 1 mM EDTA and 10 mM Tris-HCl, pH 8). The immunocomplexes were eluted with freshly made elution buffer (0.1 M NaHCO_3_ and 0.5% SDS) and incubated at 65 °C for 15 min. The crosslink was reversed by incubation at 65 °C for overnight in the presence of 250 mM NaCl. Proteins were digested by adding 20 mL of 1 M Tris-HCl, pH 6.5, 10 mL of 0.5 M EDTA, and 2 mL proteinase K (10 mg/mL) and incubated at 45 °C for 2 h. Immunoprecipitated DNA was purified using a mixture of phenol:chloroform:isoamylalcohol (25:24:1) and subsequently used for qRT-PCR with the primers listed in Table S1.

### Dual-Luciferase Assays

Coding regions of CBF3 was cloned into the pCAMBIA1304 binary vector as the effector plasmid, in which CBF3 was driven by 35S promoter. Four fragments of GA2ox7 promoter truncations were inserted into the pGreenII-0800-LUC as the reporter plasmids. All constructs were introduced into Agrobacteria strain GV3101, respectively. The pSoup-P19 helper plasmid was co-transformed with pGreenII- 0800-LUC vectors. The reporter and effector Agrobacteria were mixed with a ratio of 2:8 and infiltrated into 3-week-old *Nicotiana benthamiana* leaves. Three days after infiltration, leaf discs were collected and the luciferase activity of extracts was analyzed using a Dual-Luciferase Assay Kit (Promega) as previously described^62^. Three replicates for each co-transformation were carried out.

## Additional Information

**Accession codes:** Sequence data used in this work can be found in the GenBank libraries with the following accession numbers: *CBF1* (AT4G25490), *CBF2* (AT4G25470), *CBF3* (AT4G25480), *GID1a* (AT3G05120), *GID1b* (AT3G63010), *GID1c* (AT5G27320), *GAI* (AT1G14920), *RGA* (AT2G01570), *RGL1* (AT1G66350), *RGL2* (AT3G03450), *RGL3* (AT5G17490), *SLY1* (AT4G24210), *GA20ox1* (AT4G25420), *GA20ox2* (AT 5G51810), *GA20ox3* (AT5G07200), *GA3ox1* (AT1G15550), *GA3ox2* (AT1G80340), *GA2ox1* (AT1G78440), *GA2ox2* (AT1G30040), *GA2ox3* (AT2G34555), *GA2ox4* (AT 1G47990), *GA2ox6* (AT1G02400), *GA2ox7* (AT1G50960).

**How to cite this article**: Zhou, M. *et al*. *Arabidopsis* CBF3 and DELLAs positively regulate each other in response to low temperature. *Sci. Rep.* **7**, 39819; doi: 10.1038/srep39819 (2017).

**Publisher's note:** Springer Nature remains neutral with regard to jurisdictional claims in published maps and institutional affiliations.

## Supplementary Material

Supplementary Information
