# Supplementary material for: Arabidopsis CBF3 and DELLAs positively regulate each other in response to low temperature
Source: Sci Rep. 2017 Jan 4;7:39819. doi: 10.1038/srep39819 (PMC5209670; doi:10.1038/srep39819)
Supplement: Supplementary Information [file srep39819-s1.pdf]

Supplementary Information

Arabidopsis CBF3 and DELLAs positively regulate each other in response to low temperature

Mingqi Zhou, Hu Chen, Donghui Wei, Hong Ma, Juan Lin

State Key Laboratory of Genetic Engineering, Institute of Plant Biology, School of Life Sciences, Fudan University, Shanghai 200433, People's Republic of China

Author for correspondence: Juan Lin, Tel: +86-21-51630536

E-mail: linjuan@fudan.edu.cn

Table S1 Oligonucleotides used in this article

Name	Sequence (5'-3')	Usage
GA2ox7-L2	TAGGTATTGCCGACACCAACATTCCACAGC	EMSA
GA2ox7-L2-m	TAGGTATTGTCTATACCAACATTCCACAGC	EMSA
CBF3-HisF	CG*GAATTC*AACTCATTTTCTGCTT (*EcoR* I site italicized)	CBF3-His expression
CBF3-HisR	CG*AAGCTT*ATAACTCCATAACGATA (*Hind* III site italicized)	CBF3-His expression
CBF1-Nco	GG*CCATGG*TAATGAACTCATTTTCAGCTT (*Nco*I site italicized)	*35S::CBF1*
CBF1-Bst	GG*GGTAACC*TTAGTAACTCCAAAGCGACA (*Bst*EII site italicized)	*35S::CBF1*
CBF2-Nco	GG*CCATGG*TAATGAACTCATGTTCTGCT (*Nco*I site italicized)	*35S::CBF2*
CBF2-Bst	GC*GGTTACC*TTAATAGCTCCATAAGGACA (*Bst*EII site italicized)	*35S::CBF2*
CBF3-Bgl	GG*AGATCT*GATGAACTCATTTTCTGCTT (*Bgl*II site italicized)	*35S::CBF3*
CBF3-Bst	CC*GGTAACC*TTAATAACTCCATAACGATA (*Bst*EII site italicized)	*35S::CBF3*
GA2ox7-Sac	CG*CCGCGG*GTAAAGCTATAGTACATGATAAG (*Sac*II site italicized)	LUC assay
Basal-Pst	GG*CTGCAG*CGGTAAGACTTGTATAAATGATAGTC (*Sac*II site italicized)	LUC assay
L3-Pst	GG*CTGCAG*GCTATTTAATTAATCTCTGTTCTATCG (SacII site italicized)	LUC assay
L1+L3-Pst	GG*CTGCAG*ATTAAACCAGCCTAATTGAAACACCC (SacII site italicized)	LUC assay
L1+L3+L2-Pst	AA*CTGCAG*GTGGCCATTTATCATTGGTTAGG (SacII site italicized)	LUC assay
L1+L3+L2m-Pst	AActgcagGTGGCCATTTATCATTGGTTAGGTATTGTCTATACC (SacII site italicized)	LUC assay
RD29a-chipF	GGGCCAATAGACATGGACCGACT	ChIP-qPCR
RD29a-chipR	TCTACGCGTGTCTGTGTCGGCTTGAT	ChIP-qPCR
GAI-chipF	GCTCTGTTCAACGGCGGTG	ChIP-qPCR
GAI-chipR	GAAATTTTTCAATTACCTAATATAATG	ChIP-qPCR
GA2ox7-L1-chipF	AGCTAAAATTCCACGAAACTGGG	ChIP-qPCR
GA2ox7-L1-chipR	ATGTTCTTGTCATTGTTGGGTTTTT	ChIP-qPCR
GA2ox7-L2-chipF	AGTAGTTAGAGAGACGAATGTGCTTTG	ChIP-qPCR
GA2ox7-L2-chipR	TTAGATATGGAATGGGCTGTGGA	ChIP-qPCR
GA2ox7-L3-chipF	ATGACAAGAACATTGAAGAAGTAACA	ChIP-qPCR
GA2ox7-L3-chipR	CCGTATCGTTTTGCTTGTTGTCACTCAT	ChIP-qPCR
RGA-L5-chipF	GGTATGGGGAATAAAATTC	ChIP-qPCR
RGA-L5-chipR	CCCGATAGAAGTACAGCTC	ChIP-qPCR
Real-GA20ox1F	TTTCACCGGACGCTTCTCC	qPCR
Real-GA20ox1R	CGCAAAACCGGAAAGAAAGG	qPCR
Real-GA20ox2F	CGATCTCTCAAGCCAAGACTCG	qPCR
Real-GA20ox2R	TCGCTGACGCCATGATTG	qPCR
Real-GA20ox3F	CAACCTCTCCAAGTCCCACTCA	qPCR
Real-GA20ox3R	AGTAGCCTCCGATGCCAAGC	qPCR

Real-GA3ox1F	TCCCGGATTCTTACAAGTGGAC	qPCR
Real-GA3ox1R	GCCGGAGGAGAAGGAGCA	qPCR
Real-GA3ox2F	CCCCTCCACGATTTCCGTA	qPCR
Real-GA3ox2R	TGCGAACCACATCAACTTGG	qPCR
Real-GA2ox1F	CTTCGCTGGACCTTCATTGAC	qPCR
Real-GA2ox1R	ACAACCTCTCGTCCTCATTGTCT	qPCR
Real-GA2ox2F	GGACCAAACGGTGACGTTG	qPCR
Real-GA2ox2R	GTACTCCTCCACCGACTCACG	qPCR
Real-GA2ox3F	GGCACACCCCTGCAATTTT	qPCR
Real-GA2ox3R	CCAGAAATTTGCTCGACATTCTC	qPCR
Real-GA2ox4F	GATGGCATGTGGGTTTCTGTC	qPCR
Real-GA2ox4R	TCTCCCGTTCGTCATCACCT	qPCR
Real-GA2ox6F	CCACGCAAATCCGACAGC	qPCR
Real-GA2ox6R	GCCAAATCTCTAACCGTGCGTA	qPCR
Real-GA2ox7F	GCCATCTAACTAGTGGTGAGGAGGT	qPCR
Real-GA2ox7R	TCCCCACTCTTTCGCAGCT	qPCR
Real-GAI-F	GCTCTGTTCAACGGCGGTG	qPCR
Real-GAI-R	GAAATTTTTCAATTACCTAATATAATG	qPCR
Real-RGA-F	GATGTTGGGTTGGCACACTC	qPCR
Real-RGA-R	GACCTACCAAAACGATATATATA	qPCR
Real-RGL1-F	GTTGCTTGGATGGCAAACGC	qPCR
Real-RGL1-R	ATCATTTCATTGGCCTGACCCTG	qPCR
Real-RGL2-F	TGGCAGACGCGACCACTCAT	qPCR
Real-RGL2-R	TACTCGTTCTCTTAACTCTCAA	qPCR
Real-RGL3-F	ACCTCTAATCGCTGCATCGG	qPCR
Real-RGL3-R	CTGTTGTTACATACACACATG	qPCR
Real-GID1a-F	GATGTCTTGATTGATCGCAGGAT	qPCR
Real-GID1a-R	AGGAGGTTGCTCTTGATCTGCA	qPCR
Real-GID1b-F	CGAGAGACCACTTCGGTTATC	qPCR
Real-GID1b-R	CTAGTACGCAAACAAAGTACCATTC	qPCR
Real-GID1c-F	AAGCAGGAAGAAGAACAGTGTAGTC	qPCR
Real-GID1c-R	AGTCAAAAGCTAACGCTAGAAAGC	qPCR
Real-SLY1-F	TTCCACATGAAGCGCAG	qPCR
Real-SLY1-R	CCTAGCACGCGGATTG	qPCR
Real-CBF3F	TTCCGTCCGTACAGTGGAAT	qPCR
Real-CBF3R	AACTCCATAACGATACGTCGTC	qPCR
Real-CBF2F	CGACGGATGCTCATGGTCTT	qPCR
Real-CBF2R	TCTTCATCCATATAAAACGCATCTTG	qPCR
Real-CBF1F	GGAGACAATGTTTGGGATGC	qPCR
Real-CBF1R	CGACTATCGAATATTAGTAACTCC	qPCR
Real-RD29aF	GGGTAGAGATTATGTGGCGGAGA	qPCR

Real-RD29aR	TTCCTTTGTCGTCGTTTCCTTC	qPCR
Real-COR15aF	CTCTGCCGCCTTGTTTGC	qPCR
Real-COR15aR	CTGAGAAAGCTGCGGCGTA	qPCR
Real-KIN1F	TGCCTTCCAAGCCGGTCAGA	qPCR
Real-KIN1R	AGGCCGGTCTTGTCCTTCAC	qPCR
Real-COR47F	CCGAGCACGAGACACCAAC	qPCR
Real-COR47R	TCCACGATCCGTAACCTCTGTT	qPCR
Actin2F	TGAGAGATTCAGATGCCCAGAA	qPCR
Actin2R	TGGATTCCAGCAGCTTCCAT	qPCR

Supplementary figure legends

Fig. S1 *Ws*, *CBF1-ox*, *CBF2-ox* and *CBF3-ox* plants growing on MS plates containing 10^{-5}M GA3, 10^{-6} M GA3. Solvent (0.1% ethanol) was used as control.

Fig. S2 Relative expression levels of *CBF1*, *CBF2* and *CBF3* in *CBF1-ox*, *CBF2-ox* and *CBF3-ox* plants in *della-global* background.

Fig. S3 Comparison of phenotypes of *CBF1-ox*, *CBF2-ox* and *CBF3-ox* plants in Ws wild type and *della-global* Ler backgrounds.

Fig. S4 GFP:RGA protein level under MeJA treatment. The 8-days-old pRGA:: GFP:RGA seedlings growing on MS plates were incubated with 50 mM MeJA for the time indicated.

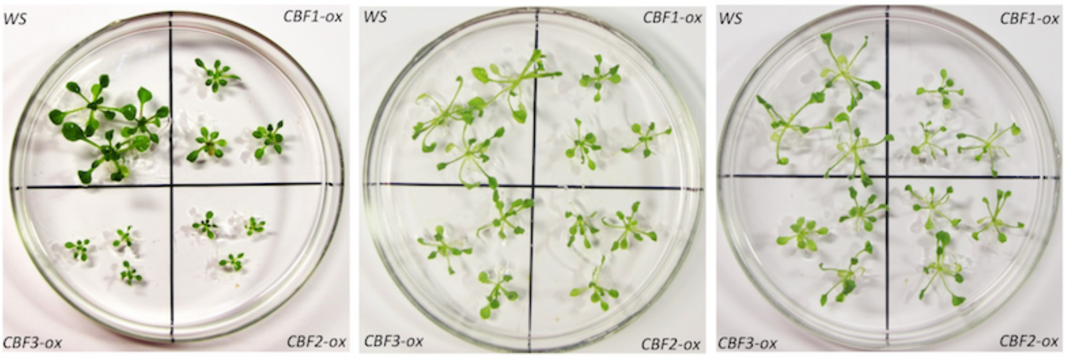

0.1 % Ethanol 10^{-5} M GA3 10^{-6} M GA3

Fig. S1

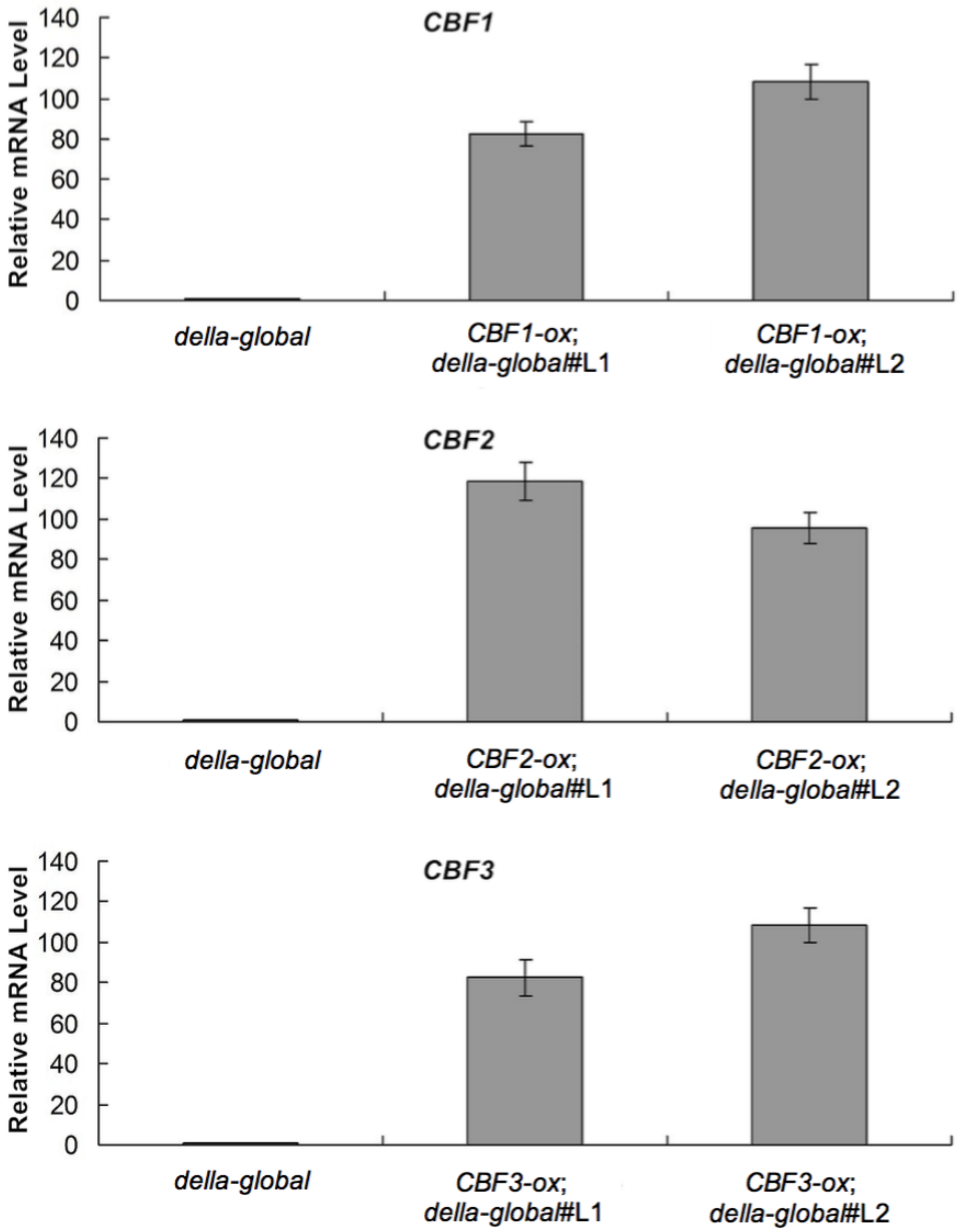
CBF1
Relative mRNA Level
140
120
100
80
60
40
20
0
della-global
CBF1-ox; della-global#L1
CBF1-ox; della-global#L2
CBF2
Relative mRNA Level
140
120
100
80
60
40
20
0
della-global
CBF2-ox; della-global#L1
CBF2-ox; della-global#L2
CBF3
Relative mRNA Level
140
120
100
80
60
40
20
0
della-global
CBF3-ox; della-global#L1
CBF3-ox; della-global#L2

Fig. S2

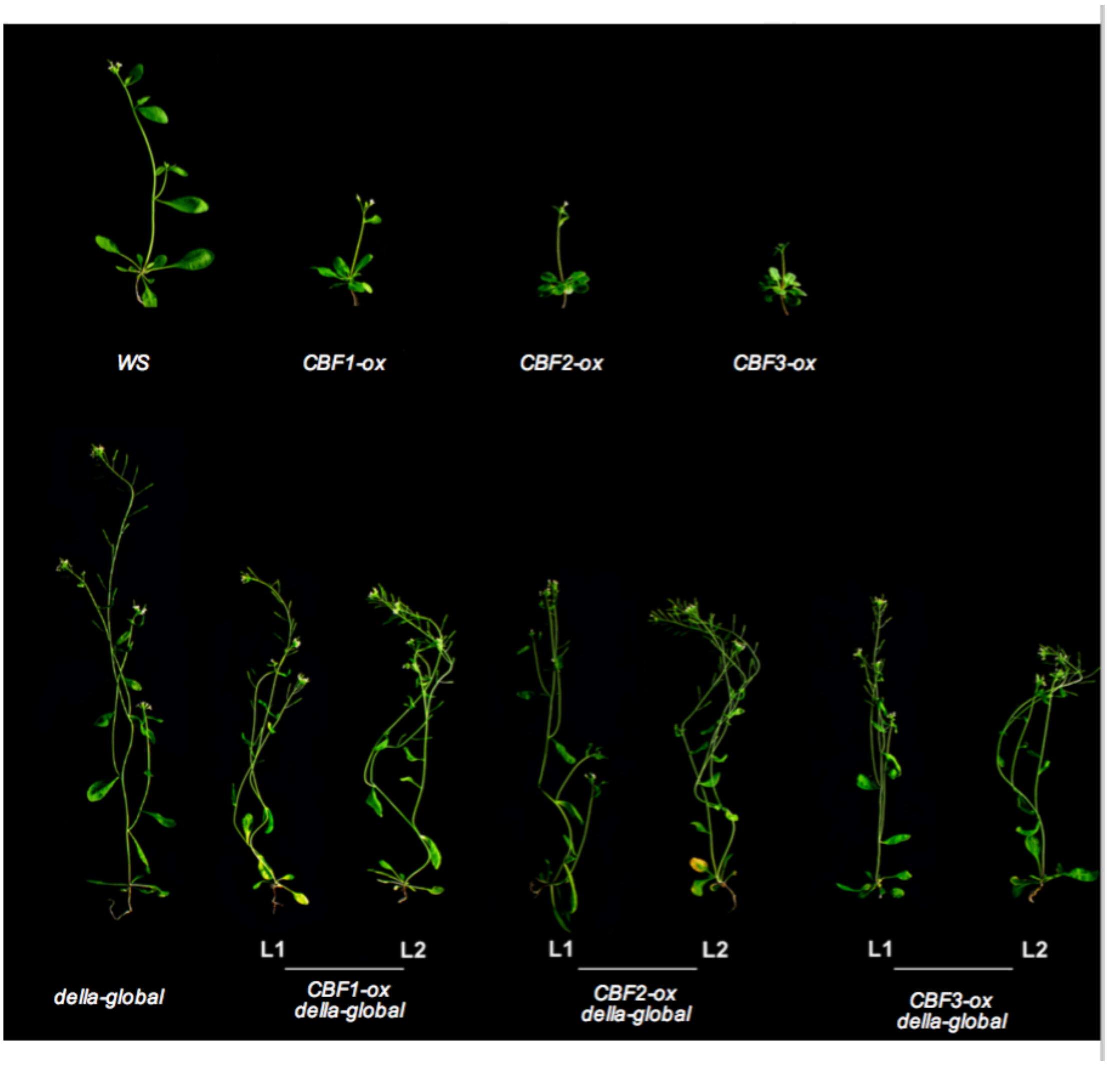
WS
CBF1-ox
CBF2-ox
CBF3-ox
della-global
L1
L2
CBF1-ox
della-global
L1
L2
CBF2-ox
della-global
L1
L2
CBF3-ox
della-global

Fig.S3

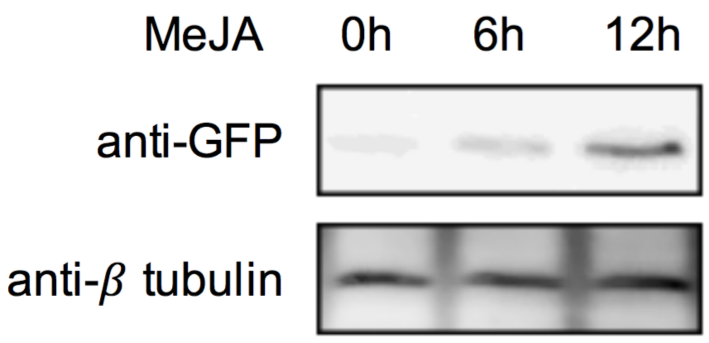

Fig. S4
